# Shrunken Social Brains? A Minimal Model of the Role of Social Interaction in Neural Complexity

**DOI:** 10.3389/fnbot.2021.634085

**Published:** 2021-06-11

**Authors:** Georgina Montserrat Reséndiz-Benhumea, Ekaterina Sangati, Federico Sangati, Soheil Keshmiri, Tom Froese

**Affiliations:** ^1^Embodied Cognitive Science Unit, Okinawa Institute of Science and Technology Graduate University, Okinawa, Japan; ^2^Computer Science and Engineering Postgraduate Program, Institute for Applied Mathematics and Systems Research, National Autonomous University of Mexico, Mexico City, Mexico

**Keywords:** agent-based modeling, social interaction, complexity, entropy, social brains, evolutionary robotics, continuous-time recurrent neural network, nonlinear time series analysis

## Abstract

The social brain hypothesis proposes that enlarged brains have evolved in response to the increasing cognitive demands that complex social life in larger groups places on primates and other mammals. However, this reasoning can be challenged by evidence that brain size has decreased in the evolutionary transitions from solitary to social larger groups in the case of Neolithic humans and some eusocial insects. Different hypotheses can be identified in the literature to explain this reduction in brain size. We evaluate some of them from the perspective of recent approaches to cognitive science, which support the idea that the basis of cognition can span over brain, body, and environment. Here we show through a minimal cognitive model using an evolutionary robotics methodology that the neural complexity, in terms of neural entropy and degrees of freedom of neural activity, of smaller-brained agents evolved in social interaction is comparable to the neural complexity of larger-brained agents evolved in solitary conditions. The nonlinear time series analysis of agents' neural activity reveals that the decoupled smaller neural network is intrinsically lower dimensional than the decoupled larger neural network. However, when smaller-brained agents are interacting, their actual neural complexity goes beyond its intrinsic limits achieving results comparable to those obtained by larger-brained solitary agents. This suggests that the smaller-brained agents are able to enhance their neural complexity through social interaction, thereby offsetting the reduced brain size.

## 1. Introduction

It is widely accepted that increased social group size is an important factor for explaining the evolution of increased brain size in primates, because of a concomitant increase in the cognitive demands posed by more complex forms of social competition and/or social bonding (Byrne, 1996; Dunbar and Shultz, 2007). However, this social brain hypothesis has come under pressure from various directions. Brain tissue is energetically expensive, and so primate brain size can only increase as an adaptive response to increased cognitive demands if such growth is enabled by a sufficiently high-quality diet. In fact, diet seems to be the primary factor that predicts brain size in primates (DeCasien et al., 2017). In this context the evolution of human brain size during the Paleolithic period can then be seen as fueled by the development of cooking, which significantly enhanced our energy intake and brought along with it an increase in cognitive and social complexity (Wrangham, 2009; Herculano-Houzel, 2016).

It might therefore be expected that during the subsequent Neolithic period, with the advent of domestication, agriculture, and settled living in ever larger groups, human brain size will have continued to increase or at least remained stable. However, the opposite tendency has been observed: during this time period around the world, human brain size underwent a notable reduction beyond that expected from an overall reduction in body size (Brown, 1987; Henneberg, 1988; Henneberg and Steyn, 1993; Brown and Maeda, 2004). In particular, reduction in cranial capacity is associated with the highest levels of population density, coinciding with the emergence of larger, socially, and economically organized ways of life that mark the start of the Neolithic period (Bailey and Geary, 2009). The overall extent of the reduction is controversial (Leach, 2003), but according to some estimates it is comparable in extent to the increase in brain size associated with previous speciation events in human evolution (Henneberg, 2006).

Although this curious fact about the most recent period of human brain evolution has so far received less attention, several hypotheses have been developed to account for it. These hypotheses can be grouped into two broad categories depending on whether they appeal to an increase in selection pressures favoring smaller brains or to a decrease in selection pressures favoring bigger brains. The former category includes selection pressure on improved brain efficiency, and more prominently the self-domestication hypothesis, which has proposed that there was an increase in selection pressure for reducing in-group competition (Hare et al., 2012; Hare, 2017), which has led to the prediction of reduced cranial capacity in Holocene humans (Cieri et al., 2014), as this is also observed in other domesticated animals (Leach, 2003).

Alternatively, it could be that domesticated ways of life placed fewer cognitive demands on individuals' brains, which would then put this hypothesis in the latter category. Indeed, rather than just reducing cognitive demands, the human sociocultural environment makes cognition more efficient and more complex (Clark, 2006; Sterelny, 2017). Human evolution is characterized by an increase in sociocultural scaffolding of learning and apprenticeship (Sterelny, 2012), and so it makes sense that the increase in social institutions in the Neolithic period permitted the human brain of “doing more with less” size (Bednarik, 2014). Hodder (2020) jokingly refers to this possibility as the “smart phones, dumb people” syndrome. To be more precise, the latest theoretical developments in cognitive science promote an embodied, embedded, extended, and enactive (“4E”) approach to the mind (Newen et al., 2018), which argues that the basis of cognition is not limited to the brain but can spread out over brain, body, and environment. On this view, cognitive processes are underdetermined by brain structure, which undermines the underlying assumption of the social brain hypothesis (Barrett et al., 2007; Barona, 2021). Living in a world of enhanced sociocultural scaffolding of cognition would permit brains to become smaller but, importantly, without a reduction in overall cognitive capacity of the appropriately scaffolded person. We could call this the social scaffolding hypothesis for decreased brain size.

Interestingly, a similar distributed cognition hypothesis has been proposed for the case of eusocial insects: in wasps, the strongest changes in brain investment, namely a reduction in central processing brain regions, accompanied the evolutionary transition from solitary to social species but did not correlate specifically with the degree of social complexity (O'Donnell et al., 2015). More generally, the evolution of insect societies appears to have occurred without the evolution of any new dedicated neural structures (Farris, 2016). Reasons for this are unclear, but a computational model suggests that there may be energetic advantages to optimizing total colony brain mass over individual brain mass (Feinerman and Traniello, 2016).

This suggests a more general hypothesis for decreased brain size, in which the key explanatory factor is not the complexity of the social world *per se*, but rather a more general principle of decreased in-group conflict and increased possibilities of relying on responsive others. In the case of insect colonies, this is achieved by the colony serving as an extended phenotype of the reproductive individuals, with most individuals being closely related to each other. In the Neolithic period human groups could no longer rely on such genetic relatedness for in-group support, but they were able to expand in size from extended family groups to large villages by developing new symbolic forms of group identification. This allowed unfamiliar individuals with a shared symbolic identity to rely on each other for support, based on early developing mutual socio-moral expectations (Jin and Baillargeon, 2017).

However, in general it remains unclear whether the brain size reduction associated with the evolution of such large, yet tightly integrated and highly coordinated in-groups is better characterized as a case of “smart phone, dumb user” or of “doing more with less.” In other words, is it the case that brain size could decrease because it was possible for brain activity to become *less complex* (“dumb”) due to a reduction of cognitive demands in the social milieu (highly dependable and organized “smart” society)? Or could it also be the case that brain size could decrease because brain activity became *more complex* due to the cognitive scaffolding provided by that reliable social milieu? That is, a smaller brain could be producing activity at least equal in complexity to a larger brain (“doing more processing with less resources”) because it became supported by extended social structures. It is difficult to empirically arbitrate between these two possibilities. One piece of evidence in support of the latter possibility is that during human evolution the brain's blood flow rate, which is indicative of levels of neural activity, has increased faster than brain size (Seymour et al., 2016). But also, more theoretical work is needed to deepen our conceptual understanding of how brains could do more with less during social interaction.

## 2. Methods

In the following we will explore these questions by employing the synthetic approach to studying adaptive behavior based on agent-based modeling, evolutionary algorithms, and dynamical systems analysis (Cliff et al., 1993; Beer, 1997; Harvey et al., 2005). We will use this approach to create a simulated “thought experiment” (Di Paolo et al., 2000) that will permit us to investigate, in the most simplified manner possible, the potential roles of brain size and sociality in the generation of an individual's neural complexity, where we will operationalize it in two ways.

First, we will consider neural complexity to be captured by Shannon entropy calculated over neural output values. While it might seem that focusing on Shannon entropy, which is maximal for uniform distributions, will bring about cognitive or behavioral randomness, there are actually good reasons to consider it as a possible measure of complexity. For one, “the principle of maximum entropy,” which states that the distribution that maximizes Shannon entropy is to be preferred (Jaynes, 1957), is found to be operational in biological systems. For instance, maximizing the neural response entropy amplifies mutual information between the brain activation and incoming stimuli (Laughlin, 1981). Furthermore, entropy has been suggested as a possible correlate of consciousness by the “entropic brain hypothesis” (Carhart-Harris, 2018).^1^ Additionally, as stated in more recent work (Candadai et al., 2019), some previous studies have associated high levels of neural entropy with enhanced cognitive performance, e.g., improved generalization in motor learning tasks (Dotov and Froese, 2018).

Second, for analysis of the results, we will complement the measure derived from information theory with a notion more closely associated with system dynamics by looking at the dimension of the attractor in the state spaces of evolved neural activity. Here, we employ the embedding dimension as a measure of the effective degrees of freedom or the complexity of the dynamics (Stam, 2005) of the corresponding agent's neural activity (neural states). In order to calculate it, we follow standard practices of nonlinear time series analysis (Kodba et al., 2005; Perc, 2006; Froese et al., 2013), by: (1) using mutual information (MI) to estimate a proper embedding delay τ (Fraser and Swinney, 1986), and (2) using the false nearest neighbor (FNN) method to determine a proper embedding dimension *m* (Kennel et al., 1992).

Research (Froese and Di Paolo, 2010; Froese et al., 2013; Campos and Froese, 2017; Candadai et al., 2019; Reséndiz-Benhumea et al., 2020) suggests that in the absence of in-group competition,^2^ it is easier to evolve increased complexity of neural activity in close interaction with other agents, than to do so alone by increasing the intrinsic complexity of the neural network architecture. In the present study, we extend these findings by focusing on the role of brain size in generating complexity of neural activity in solitary and social conditions. Our aim is to show that it is possible for social agents with a smaller neural network to exhibit at least the same complexity of neural activity as a solitary agent with a larger neural network.

### 2.1. Model

The implementation of the proposed model is based on the Candadai et al. (2019) model. Here, we evolved agents to maximize their neural entropy in individual and social scenarios using a smaller (2-neuron model) and a larger (3-neuron model) neural networks.

#### 2.1.1. Simulated Agents and Environment

Agents have circular bodies, with a radius of 4 units. Each of them is provided with: two acoustic sensors, which are symmetrically positioned in its frontal side at ±45° with respect to its central axis; an acoustic emitter, which is located in its body's center; and, two motors, which are driving two wheels in its left and right sides, respectively, that enable displacement in a 2-dimensional environment. The environment consists of an empty open-ended arena.

Each agent emits an acoustic signal and senses the strength of another agent's signal, which experiences attenuation due to distance and “self-shadowing” mechanism (see Supplementary Material for details).

#### 2.1.2. Neural Architecture

**Figure 1:**
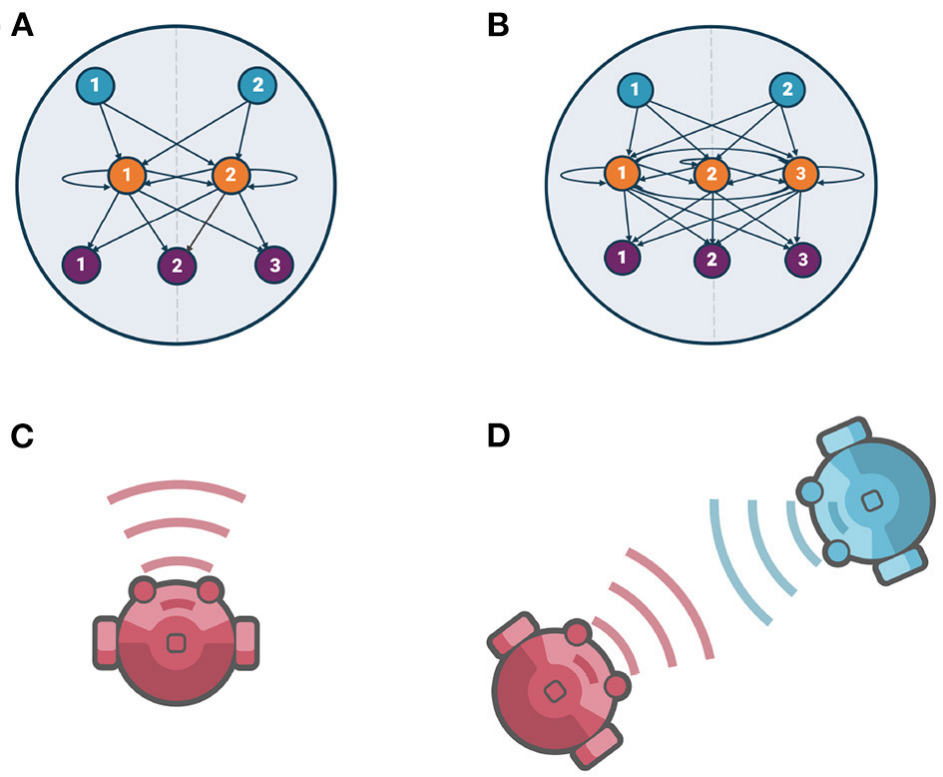
Agents' neural architecture and evolutionary conditions, **(A)** 2-neuron model neural architecture, **(B)** 3-neuron model neural architecture. In both of them, sensor nodes, neurons (fully recurrently connected neurons), and actuator nodes are shown by blue, orange, and purple nodes, respectively. **(C)** Evolving solitary agents [Individual Evolution (IE)], **(D)** Evolving interacting pair of agents [Social Evolution (SE)].

The agent's neural architecture consists of three fully connected layers: sensor layer, neuron layer, and actuator layer. In this work, we study the comparison between 2-neuron and 3-neuron models. The main difference between them is the number of neurons in their neuron layer (two and three neurons, respectively), while their sensor and actuator layers are identical (see Figure 1).

The sensor layer consists of two sensor nodes with a sigmoidal activation function. The inner neuron layer is modeled as a continuous-time recurrent neural network (CTRNN) (Beer, 1995). In this layer, we are implementing two brain architectures: the use of two fully recurrently connected neurons for the 2-neuron model as shown in the Figure 1A, which corresponds to a 2-dimensional dynamical system; and, the use of three fully recurrently connected neurons for the 3-neuron model as shown in the Figure 1B, which corresponds to a 3-dimensional dynamical system. In both architectures, each neuron's activity is governed by the standard CTRNN state equation. The actuator layer consists of three actuator nodes, two correspond to the left and right motors for agent locomotion and one corresponds to the acoustic signal emitter for modulating the strength of the emitted signal. All actuator nodes are sigmoidal units with no internal state. Agent locomotion is the result of the effective control of the two motors, where net linear velocity is given by the average of the outputs of the two actuator nodes corresponding to the right and left motors; and, the angular velocity, which refers to how fast an agent rotates, is given by their difference divided by the radius of the agent. The equations for each layer are provided in the Supplementary Material.

#### 2.1.3. Performance Measure

The measure that we use for evaluating agent's performance is its neural entropy. In particular, we select simple multi-dimensional Shannon entropy calculated on neural output values, as in the Candadai et al. (2019) model (see Supplementary Material for details).

#### 2.1.4. Genetic Algorithm

A real-valued genetic algorithm was used to optimize the parameters of the agents' neural controllers (i.e., the connection weights, the time-constants, the biases, and the gains) to maximize their neural entropy. It is important to highlight that no particular task was explicitly optimized. For the 2-neuron and 3-neuron models, each agent had 20 and 30 parameters, respectively. These parameters were encoded as a real-valued genotype, where each value was contained in the interval [-1,1] and scaled during simulation to specific parameter ranges, which are provided in the Supplementary Material.

The agents were evaluated in each of the 4 independent trials and their neural outputs recorded in order to calculate their normalized neural entropy. Each trial lasted 200 simulation seconds at a step size of 0.1.

Upon ranking the population according to fitness values, the new population was generated by keeping an elite population of the top 4% of the existing solutions and by mutating and crossing over these elite individuals to get the rest of the new solutions. Mutation was performed by adding zero-mean Gaussian mutation noise with a variance of 0.1 to the solutions and crossover involved swapping each parameter between a pair of solutions with a probability of 0.1.

#### 2.1.5. Experimental Setup

For both brain sizes (2-neuron and 3-neuron models) we evolved agents in two different evolutionary conditions: individual and social. Here, we describe the implementation details for both scenarios.

1. **Individual Evolution (IE):** We performed 10 independent runs with an initial random population of 96 solitary agents, i.e., without an agent partner as shown in Figure 1C. The parameters for each agent were encoded as a single genotype (one solution). The solitary agents were not sensing any input, neither from another agent nor from the environment. For each trial, the agent's initial position was set at coordinates (0, 0). The agent's heading direction was initialized to the right. The population was evolved for 2000 generations to maximize the neural entropy of each solitary agent.
2. **Social Evolution (SE):** We performed 10 independent runs with an initial random population of 96 pairs of agents that were able to interact with each other as shown in Figure 1D. The parameters for each pair of agents were encoded as a single genotype (one solution) and were evolved together during all generations. For each trial, there was a fixed initial position for each pair of agents: the first agent was always positioned at coordinates (0, 0), while the second agent was placed 20 units of distance from the first one, just varying their relative angle as [0, *π*/2, *π*, 3*π*/2], respectively. Furthermore, both agents' heading direction was initialized to the right. The population was evolved for 2000 generations to maximize the neural entropy of each pair of agents.

## 3. Results

In this section, we present the results for the best agents in 2-neuron and 3-neuron models from the corresponding runs. We focus on the comparison between 3-neuron agents evolved in solitary environment (IE) and 2-neuron agents evolved in social environment (SE).

### 3.1. Agent Behavior

**Figure 2:**
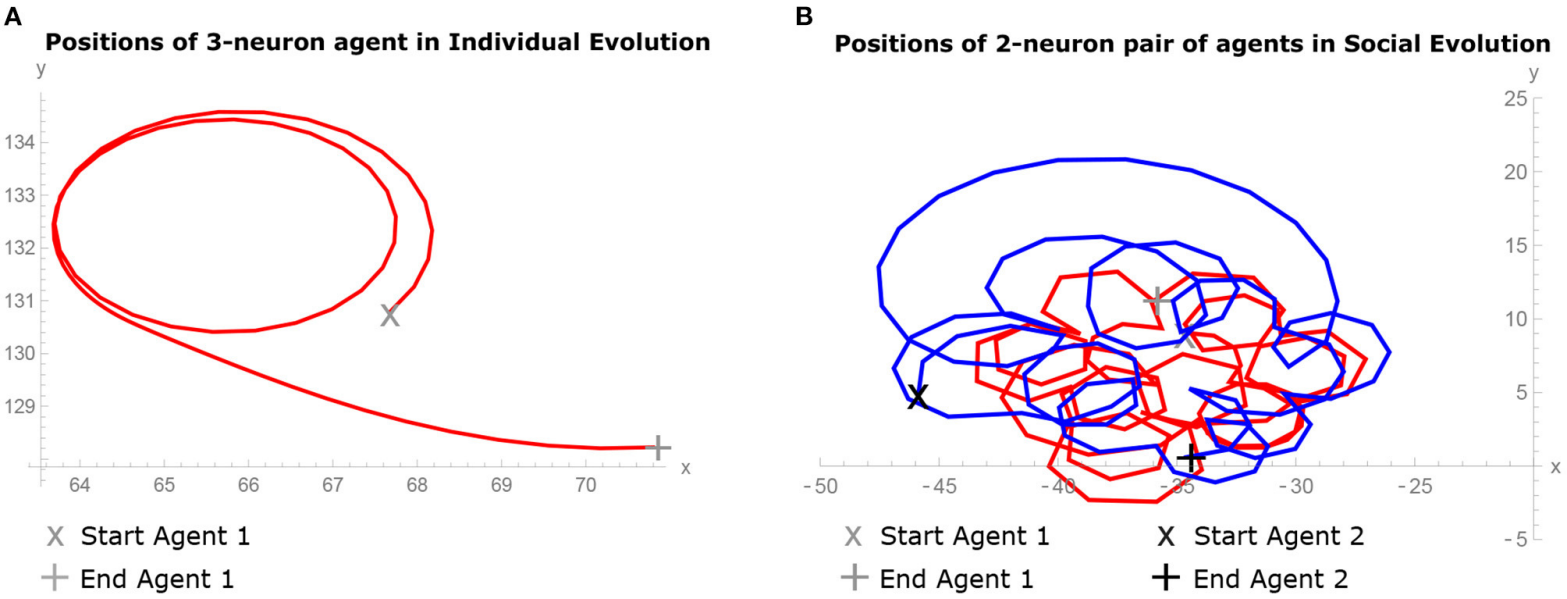
Behavior of agents during the last 10 simulation seconds of the trial. **(A)** Trajectory of the best 3-neuron agent evolved in Individual Evolution (IE) condition from the best run, showing simple loops movement. **(B)** Trajectories of the best pair of 2-neuron agents evolved in Social Evolution (SE) condition from the best run, showing spiralling nested pairwise loops movement.

Figure 2A shows the trajectory of the best agent evolved in the IE condition in 3-neuron model from the best run. Figure 2B shows the trajectory of the best pair of agents evolved in the SE condition in 2-neuron model from the best run. In the comparison of both images, it can be observed that the solitary agent exhibits less complex behavior than the pair of agents in interaction. In the former case, the agent is moving in simple loops, while in the latter, the agents are enhancing each other behavior by displaying spiralling nested pairwise loops movement.

### 3.2. Statistical Analysis

**Figure 3:**
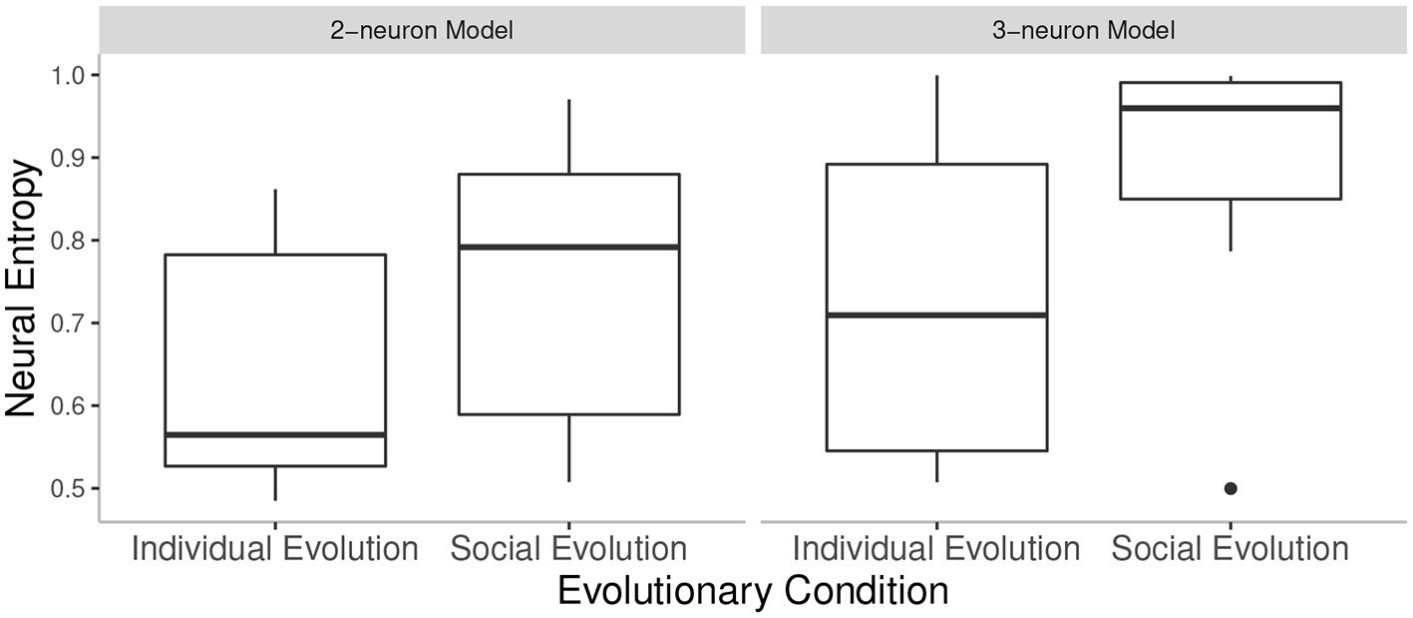
Neural entropy values in all conditions tested: values from the best agent (IE) or agent pair (SE) in the last generation in 10 runs of each condition. Note that the outlier in SE of 3-neuron model was not removed from the analysis.

In order to capture the statistical differences between the conditions tested, we compared the means of neural entropy of evolved pairs of agents in the SE conditions against the neural entropy of evolved agents in the IE conditions, between 2-neuron and 3-neuron models. Specifically, we took the neural entropy values from the best agent pair or the best agent in the last generation of all 10 independent runs in each condition. There was a significant main effect of condition *F*(1, 36) = 6.55, *p* < 0.05 and a significant main effect of the number of neurons *F*(1, 36) = 4.62, *p* < 0.05 but no significant interaction effects. That is, neural entropy was higher in SE condition than in IE condition, and higher in 3-neuron than in 2-neuron model but these factors did not affect each other (see Figure 3). Social interaction between the larger-brained agents did not lead to a larger (or smaller) neural entropy gain compared to the smaller-brained agents.

Since we were specifically interested in comparing neural entropy between agents evolved in social interaction (SE) in a 2-neuron model and agents evolved in isolation (IE) in a 3-neuron model, we also conducted a Bonferroni-corrected *post-hoc t*-test between these two conditions. The difference was not significant, *p* = 0.79.

### 3.3. Nonlinear Time Series Analysis

We performed the nonlinear time series analysis of the evolved agents' neural activity (neural states) to determine their proper embedding delay τ using MI method, and then their proper embedding dimension *m* using FNN method (see Supplementary Material for details).

We distinguished two different testing modes for obtaining the time series of the evolved agents' (in IE or SE, respectively) neural activity: (1) decoupled, when the evolved agent is tested in isolation (Input = 0); and (2) coupled, when the evolved agent is tested in the presence of an interactive partner. Furthermore, we only considered the neural states of neuron 1, trial 1, of the best agents from each run (10 runs), from 2-neuron and 3-neuron models, correspondingly, to obtain their embedding dimension.

**Figure 4:**
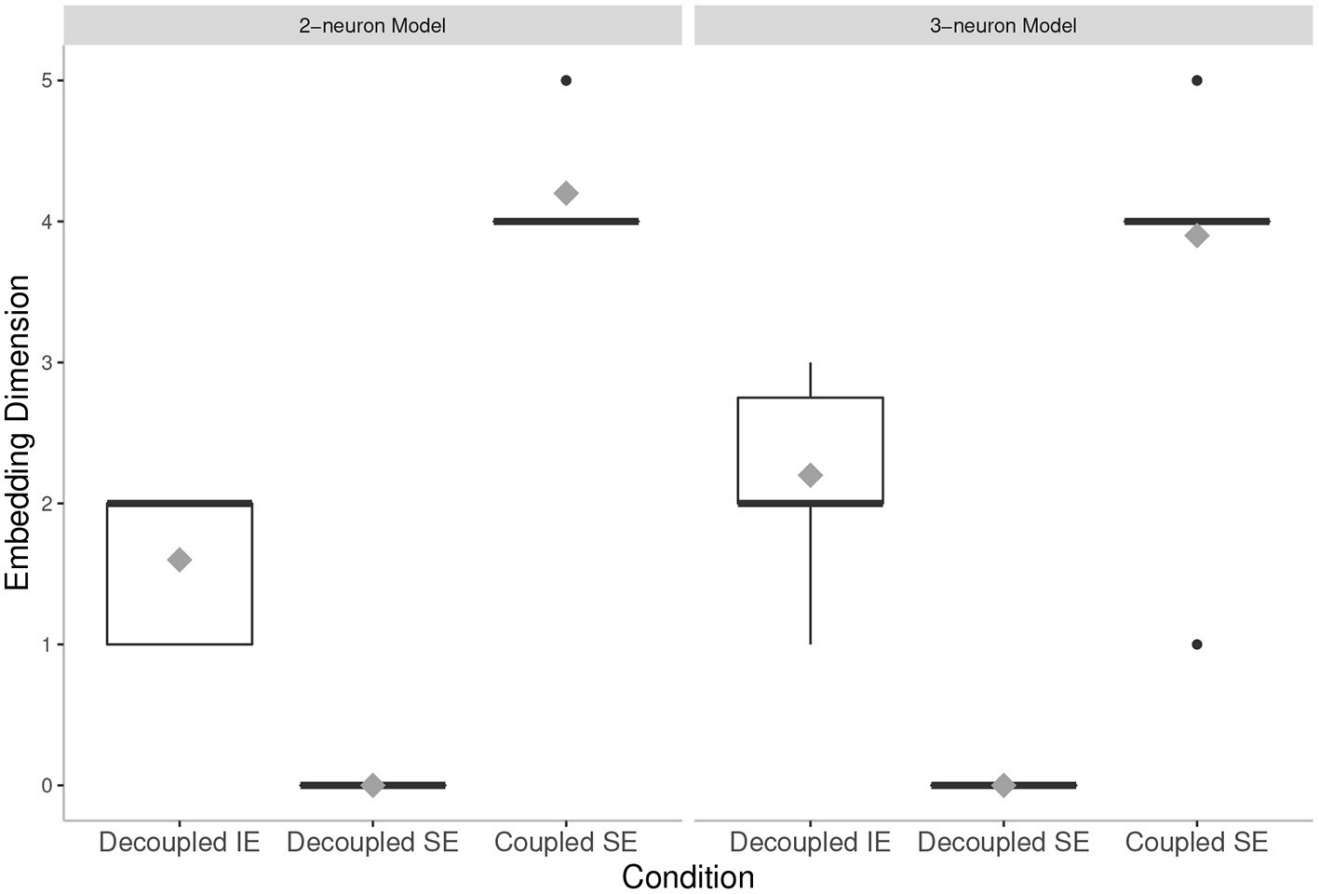
Embedding dimension in all conditions tested: The mean values are presented by gray diamonds. The mean value of the Coupled Social Evolution (Coupled SE) condition in 2-neuron model is higher than the mean value of the Decoupled Individual Evolution (Decoupled IE) condition in 3-neuron model. Note that, for both models, in the Coupled SE condition, the mean values are higher than their intrinsic dimensional limitations (i.e., 2-dimensional and 3-dimensional systems, for the cases of 2-neuron and 3-neuron models, respectively). On the other hand, for both models, in the Decoupled Social Evolution (Decoupled SE) condition, the mean values are lower than their intrinsic dimensional limitations.

Our results (see Figure 4) confirmed our hypothesis:

1. By comparing the mean embedding dimension of the neural states of the coupled 2-neuron agent evolved in SE condition (2-neuron model, Coupled SE) and the decoupled 3-neuron agent evolved in IE condition (3-neuron model, Decoupled IE), we found that the former was higher dimensional than the latter, thereby demonstrating that social smaller-brained agents have at least equivalent (i.e., equal or more) degrees of freedom of neural activity than isolated larger-brained agents.
2. Importantly, we found that this increase in degrees of freedom of neural activity in coupled socially evolved agents does not depend in their intrinsic complexity, as when being decoupled, their embedding dimension is lower than what can be achieved. This suggests, in line with previous work (Candadai et al., 2019; Reséndiz-Benhumea et al., 2020), that agents in interaction are enhancing each other's neural complexity.

## 4. Discussion

In this paper, we have investigated the idea that brain size could decrease because agents could reliably take advantage of social interaction. A key open question in this regard is whether this reduction in brain size is made possible because of decreased cognitive demands (“smart phone, dumb user”), or because of increased cognitive scaffolding (“doing more with less”).

By performing a statistical analysis, we found that smaller-brained social agents are able to exhibit comparable levels of neural complexity, as larger-brained solitary agents. This is in line with the idea that the brain of an agent in a reliable social setting can do more with less, and is consistent with previous findings (Candadai et al., 2019; Reséndiz-Benhumea et al., 2020). Then, by performing a nonlinear time series analysis, we found that the embedding dimension of the neural states of the decoupled neural network is lower when agents evolved under social condition, which seems to be more in line with the idea of the brain as a “dumb user.” Therefore, in a way, both possibilities can co-exist depending on whether we focus on the topological structure (i.e., number of neurons) or the state dynamics (i.e., degrees of freedom) of the brain: during evolution of sociality an individual's brain topological structure can become simplified, while its state dynamics can become more complex at the same time.

An important implication of this computational proof of concept for the science of brain evolution, whether in humans or social insects, is that care should be taken when inferring cognitive capacities from brain size. The coupled brain, as part of a whole body and environment system, will exhibit neural dynamics that are underdetermined by the structure of the brain.

There are several limitations to our model that need to be taken into consideration. First, our simulation involved separate evolutionary runs for different brain sizes and different conditions rather than integrating these factors within the same evolution. An alternative setup would be evolving a population of solitary large-brained agents and then transitioning them to a social smaller-brained population. It would be interesting to examine whether in such a scenario the same types of neural state spaces would be observed. Conceivably, the process of first evolving individual agents with a larger, more complex brain would be in tension with its later simplification once a social dimension is introduced and brain size is reduced.

Second, an important characteristic of the brain neural complexity lies in its modular architecture in which the interplay between specialized (i.e., segregated) and integrated neuronal units results in variety and flexibility of cognition. In this regard, the use of Shannon entropy for measuring complexity is limited as it does not allow for quantification of such functional integration among differential neural activities. This can be addressed by exploring the use of more comprehensive measures of complexity (Tononi et al., 1994), thereby capturing the utility of such potential interplay between agent's neural units on their evolved behavior.

Third, the lack of input to the agents in isolated condition was an unrealistic impoverishment in that one could attribute the higher neural complexity in social than individual evolution to a richer input provided to the former. This is consistent with our previous result (Reséndiz-Benhumea et al., 2020) that evolving an isolated agent in the presence of a non-interactive “partner” can also lead to high neural complexity. The point here, however, is not to exclude other factors that might enable neural complexity.

Fourth, social interaction simulated in our model is of a relatively simple kind: the agents were evolved always in the same pairs. It might be argued that our results are due to the availability of the same reliable partner and that a more realistic scenario in which they would have to interact with multiple, more unpredictable partners would not be possible with a smaller brain. Furthermore, brain size undoubtedly did increase over most of human evolution (Herculano-Houzel, 2016). We would like to point out that our work is not intended to deny this phenomenon but rather to put a more nuanced question into the spotlight. Namely, that it needs to be investigated what kind of social (and environmental) conditions lead to what type of consequences for brain structure and activity. Future work could explore how our findings scale up to larger groups and different kinds of social couplings.

Finally, the agents in our simulation did not have any specific task they were required to solve as we optimized neural complexity directly. This might make conclusions about *task-related* neural complexity as opposed to *task-independent* complexity not entirely justified. However, related work by Nagar et al. (2019) that did include a behavioral task found results similar to ours.

In addition to raising new modeling questions, our work leads to novel hypotheses that could be tested in experimental work. For instance, it is possible that even though social insects tend to have smaller brains than solitary insects, the individuals in a colony may nevertheless exhibit more complex neural activity.

## Data Availability Statement

The raw data supporting the conclusions of this article will be made available by the authors, without undue reservation.

## Author Contributions

All authors listed have made a substantial, direct, and intellectual contribution to the work, and approved it for publication.

## Conflict of Interest

The authors declare that the research was conducted in the absence of any commercial or financial relationships that could be construed as a potential conflict of interest.

## Footnotes

^1^We admit that generalizability of our results needs to be checked with other possible measures of neural complexity. However, given that a choice of a particular measure is in itself a challenging endeavor and each of the measures has advantages and disadvantages for trying to capture any particular phenomenon (cf. a comparison of 12 different entropy measures for just anesthesia Liang et al., 2015), we believe using the most straightforward approach for understanding the fundamental conceptual issues we tackle here is a good starting point.

^2^In the studies mentioned above and the present study, this feature is built-in given that selection pressure acts on pairs of agents together, rather than on individuals.
