# Supplementary material for: Shrunken Social Brains? A Minimal Model of the Role of Social Interaction in Neural Complexity
Source: Front Neurorobot. 2021 Jun 11;15:634085. doi: 10.3389/fnbot.2021.634085 (PMC8226032; doi:10.3389/fnbot.2021.634085)
Supplement: Supplementary file 1 [file Data_Sheet_1.pdf]

Supplementary Material

This supplementary material document contains additional details of the methods section, including a description of the performed analysis.

1 METHODS

Our code for simulations is available on GitHub: `https://github.com/oist/ecsu-dyadic_interaction`

1.1 Simulated agents and environment

The strength of an agent's acoustic signal is attenuated in two different ways:

- **Attenuation due to distance:** The strength of the acoustic signal (or sound intensity) decays inversely proportional to the square of the distance from the sound source, i.e., it obeys the inverse square law. It will be maximum and equal to that of the emitted strength at a distance equal to the $2R$ between the center of the agents.
- **Attenuation due to "self-shadowing" mechanism:** The "self-shadowing" mechanism refers to the linear attenuation of the strength of the acoustic signal, which is proportional to the distance traveled by the signal within the agent's body, i.e., the shielded distance, D_{sh}. The range of D_{sh} goes from 0, when there is a direct line between the sound source and the sensor, to $2R$, when the sensor is directly opposed to the sound source. This attenuation is a natural consequence of the agent's embodiment (Di Paolo, 2000). The equations to calculate the shielded distance, D_{sh}, are available in the Supplementary Material of the Candadai et al. (2019) model.

Thus, the sensory input for each agent's acoustic sensor (i.e., the attenuated strength of another agent's acoustic signal) is obtained by first, applying the inverse square law to the sound intensity at the position of the sensor, and then multiplying it by an attenuating factor that goes linearly from 1, when $D_{sh} = 0$, to 0.1, when $D_{sh} = 2R$ (Di Paolo, 2000).

Agents are able to navigate freely in the environment except when they are involved in a collision. Collisions are modeled as point elastic, i.e., no effect in the agents' angular velocity (frictionless bodies) and the entire system's energy is conserved (no loss of energy). Agent bodies are considered as identical, so that when they are colliding, the result is the instantaneous exchange of their velocity vectors. Due to lack of inertia, agents take back control of their movement immediately after colliding (Di Paolo, 2000).

1.2 Neural architecture

The output of the **sensor layer** nodes is given by:

$$o_s = g_s \sigma(I_s + \theta_s) \tag{S1}$$

where $\sigma(x) = 1/(1 + e^{-x})$ is the sigmoidal activation function, I_s is the sensory input, θ_s is the bias, and g_s is the gain. Both sensor nodes share common bias and gain.

In both 2-neuron and 3-neuron architectures, each **inner layer** neuron's activity is governed by the following state equation:

$$\tau_i \frac{dy_i}{dt} = -y_i + \sum_{j=1}^{N} w_{ij}\sigma(y_j + \theta_j) + \sum_{s=1}^{2} w_{is} o_s \tag{S2}$$

where dy_i/dt refers to the rate of change of internal state y_i of neuron i based on a time constant τ_i. The rate of change dy_i/dt depends on three values: the current state of the neuron, the weighted sum of outputs from all N neurons (for the 2-neuron model $N = 2$ and for the 3-neuron model $N = 3$) in the network, and the total external input. The input from other neurons is obtained by weighting their output with weights w_{ij} from neuron j to i. The output of each neuron based on its internal state is given by a sigmoidal activation function $\sigma(y_j + \theta_j)$ where θ_j refers to the neuron's bias. The total external input received by the neuron is given by the weighted sum of the sensory input with weights $w_i s$ from sensor node s to neuron i and o_s is the sensory output from each sensor node. All neurons share common time-constant and bias.

The input to the **actuator layer** is a weighted sum of the neurons' outputs. The output of each of the actuator layer nodes i, m_i, is given by:

$$m_i = g_m \sigma\left(\sum_{n=1}^{N} w_{ni} * o_n + \theta_i\right) \tag{S3}$$

where o_n is the output of the corresponding neuron n, that is weighted by w_{ni}, θ_i is the bias, and g_m is the gain. All actuator nodes share common bias and gain.

1.3 Performance measure

Neural complexity is calculated by creating a 2-dimensional or 3-dimensional (depending on the number of neurons in the inner layer) histogram of neural output values in a given trial. The neural outputs are obtained from a sigmoid function, therefore, they are bounded in the range $[0, 1]$. The output space is divided into 100 bins along each dimension, i.e., totaling ten thousand bins for the 2-neuron model (2-dimensional output space) and one million bins for the 3-neuron model (3-dimensional output space).

Then, the bins are filled with data points collected for each trial and the probability of the neural activity being in a given bin $[i, j]$, $p_{i,j}$ or $[i, j, k]$, $p_{i,j,k}$, respectively, is given by the number of points in that bin divided by the total number of points in the trial ($N = 2000$).

From these probabilities, the neural entropy H is given by:

$$H = \sum_{i=1}^{100}\sum_{j=1}^{100} -p_{ij} log_2(p_{ij}) \tag{S4}$$

$$H = \sum_{i=1}^{100}\sum_{j=1}^{100}\sum_{k=1}^{100} -p_{ijk} log_2(p_{ijk}) \tag{S5}$$

for the 2-neuron and 3-neuron models, respectively.

Next, the neural entropy is normalized to be in the range $[0, 1]$ by dividing by the maximum neural entropy that can be achieved in the given dimension and with the given number of data points., i.e., $log_2(total\_number\_of\_fillable\_bins)$, where $total\_number\_of\_fillable\_bins =$

$min(total\_number\_of\_bins, total\_number\_of\_data\_points)$. This is achieved when the data points for a specific trial are uniformly populated among the bins. Hence, the normalized neural entropy is given by:

$$\hat{H} = H/log_2(total\_number\_of\_fillable\_bins) \tag{S6}$$

For both 2-neuron and 3-neuron models, the total number of data points is 2000 per trial (i.e., one data point per step size 0.1 for 200 simulation seconds). Since for both cases, the number of data points in each trial is less than the total number of bins, we have that $total\_number\_of\_fillable\_bins = 2000$. Thus, the normalized neural entropy per trial is given by:

$$\hat{H} = H/log_2(2000) \tag{S7}$$

In contrast to the Candadai et al. (2019) implementation, we calculate neural entropy measure for each experimental trial separately. The individual fitness value for an agent is calculated as the average normalized neural entropy of all trials. In the case of interacting pairs of agents, the fitness value for each pair is calculated as the average of their individual fitness values.

1.4 Genetic algorithm

We used the following parameter ranges to scale genotype values: for sensor and actuator nodes, the gains were scaled in the interval [1,20] and [1,5], respectively; for neurons, the time-constants were scaled in the interval [1,2]; all connection weights were scaled in the interval [-8,8] and all biases were scaled in the interval [-3,3].

1.5 Nonlinear Time Series Analysis

Nonlinear time series analysis is a useful approach to understanding the underlying dynamics of a system based solely on its realizations (e.g., time series observations) and without direct/explicit knowledge to its (unknown) properties. It is a *top-down* approach in which one utilizes the system's available observations to realize its potential state space and attractor(s) (Stam, 2005).

Let $\{x_0, x_1, x_2, ..., x_t, ..., x_n\}$ be a time series associated with a system in which x_t denotes the system's output at time t. Such time series along with *delay coordinate embedding* (Takens, 1981; Kodba et al., 2005; Perc, 2006) procedure can be utilized to reconstruct the system's attractor $\mathbf{p}(t) = (x_t, x_{t+\tau}, x_{t+2\tau}, ..., x_{t+(m-1)\tau})$, where τ and m are the embedding delay and the embedding dimension, respectively.

To estimate the optimal embedding delay τ, Fraser and Swinney (1986) used the position at which x_t and $x_{t+\tau}$ attained their first minimum mutual information (MI) i.e., the minimum amount of information that the state x_t provided about the state $x_{t+\tau}$ (Kodba et al., 2005; Perc, 2006).

For estimating a proper embedding dimension m, Kennel et al. (1992) introduced the false nearest neighbor method (FNN). The main idea of this method consists of minimizing the fraction of points having a false nearest neighbor through the choice of a sufficiently large embedding dimension m.

In the present study, we were interested in determining the above embedding dimension m for the time series of the evolved agents' neural activity (neural states) in different conditions. We achieved this objective as follows:

1. We obtained the time series of neural activity in both, 2-neuron and 3-neuron models, for the following conditions:

- **Decoupled Individual Evolution (Decoupled IE):** From the best evolved agents (agent 1) of each run (10 runs) in IE condition, we tested them in isolation for 4 trials. Then, for determining the embedding dimension m, we used the time series of neural states of neuron 1, trial 1, from each isolated agent (agent 1).
- **Decoupled Social Evolution (Decoupled SE):** From the best evolved pairs of agents (agent 1 and agent 2) of each run (10 runs) in SE condition, we tested each agent 1 in isolation for 4 trials. Then, for determining the embedding dimension m, we used the time series of neural states of neuron 1, trial 1, from each isolated agent (agent 1).
- **Coupled Social Evolution (Coupled SE):** From the best evolved pairs of agents (agent 1 and agent 2) of each run (10 runs) in SE condition, we tested each pair, consisted of agent 1 and agent 2, in interaction for 4 trials. Then, for determining the embedding dimension m, we used the time series of neural states of neuron 1, trial 1, from each agent 1.

2. In order to estimate the optimal embedding delay τ using MI, we employed the nonlinear time series analysis "*mutual.exe*" program provided by Perc (2006). It requires the following parameters to be set by the user: number of data points, number of bins, and maximal embedding delay. We estimated the proper number of bins using the *Freedman–Diaconis rule* (Freedman and Diaconis, 1981):

$$Bins = \frac{\|max(x) - min(x)\|}{2\frac{IQR(x)}{\sqrt[3]{n}}} \tag{S8}$$

where $IQR(x)$ is the interquartile range of the data and n is the number of observations in the sample x.

3. In order to determine the proper embedding dimension m using the FNN with Euclidean metric (Kennel et al., 1992), we employed the *NoLiTSA Python module* provided by Mannattil (2018), where we specified the optimal embedding delay τ previously estimated.

REFERENCES

Candadai, M., Setzler, M., Izquierdo, E. J., and Froese, T. (2019). Embodied dyadic interaction increases complexity of neural dynamics: A minimal agent-based simulation model. *Frontiers in Psychology* 10:540. doi:10.3389/fpsyg.2019.00540

Di Paolo, E. A. (2000). Behavioral coordination, structural congruence and entrainment in a simulation of acoustically coupled agents. *Adaptive Behavior* 8, 27–48. doi:10.1177/105971230000800103

Fraser, A. M. and Swinney, H. L. (1986). Independent coordinates for strange attractors from mutual information. *Physical Review A* 33, 1134–1140. doi:10.1103/PhysRevA.33.1134

Freedman, D. and Diaconis, P. (1981). On the histogram as a density estimator: L2 theory. *Probability Theory and Related Fields* 57, 453–476. doi:10.1007/BF01025868

Kennel, M. B., Brown, R., and Abarbanel, H. D. I. (1992). Determining embedding dimension for phase-space reconstruction using a geometrical construction. *Physical Review A* 45, 3403–3411. doi:10.1103/PhysRevA.45.3403

Kodba, S., Perc, M., and Marhl, M. (2005). Detecting chaos from a time series. *European Journal of Physics* 26, 205–215. doi:10.1088/0143-0807/26/1/021

Mannattil, M. (2018). NoLiTSA (NonLinear Time Series Analysis) Python module. *GitHub repository* URL: `https://github.com/manu-mannattil/nolitsa`

Perc, M. (2006). Introducing nonlinear time series analysis in undergraduate courses. *Fizika A* 15, 91–112

Stam, C. J. (2005). Nonlinear dynamical analysis of EEG and MEG: Review of an emerging field. *Clinical Neurophysiology* 116, 2266–2301. doi:10.1016/j.clinph.2005.06.011

Takens, F. (1981). Detecting strange attractors in turbulence. In *Dynamical Systems and Turbulence, Warwick 1980. Lecture Notes in Mathematics*, eds. D. Rand and L. S. Young (Berlin, Heidelberg: Springer), vol. 898. 366–381. doi:10.1007/BFb0091924
